# Natural Herbal Non-Opioid Topical Pain Relievers—Comparison with Traditional Therapy

**DOI:** 10.3390/pharmaceutics14122648

**Published:** 2022-11-29

**Authors:** Dalia M. Kopustinskiene, Urte Bernatonyte, Yuliia Maslii, Nataliia Herbina, Jurga Bernatoniene

**Affiliations:** 1 Institute of Pharmaceutical Technologies, Faculty of Pharmacy, Medical Academy, Lithuanian University of Health Sciences, Sukileliu pr. 13, LT-50161 Kaunas, Lithuania; 2 Faculty of Pharmacy, Medical Academy, Lithuanian University of Health Sciences, Sukileliu pr. 13, LT-50161 Kaunas, Lithuania; 3 Department of Drug Technology and Social Pharmacy, Faculty of Pharmacy, Medical Academy, Lithuanian University of Health Sciences, Sukileliu pr. 13, LT-50161 Kaunas, Lithuania; 4 Department of Industrial Technology of Drugs, National University of Pharmacy, 61002 Kharkiv, Ukraine

**Keywords:** pain control, alternative medicine, therapy

## Abstract

Pain is the predominant symptom of many clinical diseases and is frequently associated with neurological and musculoskeletal problems. Chronic pain is frequent in the elderly, causing suffering, disability, social isolation, and increased healthcare expenses. Chronic pain medication is often ineffective and has many side effects. Nonsteroidal over-the-counter and prescription drugs are frequently recommended as first-line therapies for pain control; however, long-term safety issues must not be neglected. Herbs and nutritional supplements may be a safer and more effective alternative to nonsteroidal pharmaceuticals for pain management, especially when used long-term. Recently, topical analgesic therapies have gained attention as an innovative approach due to their sufficient efficacy and comparatively fewer systemic side effects and drug–drug interactions. In this paper, we overview the main natural herbal pain relievers, their efficacy and safety, and their potential use as topical agents for pain control. Although herbal-derived medications are not appropriate for providing quick relief for acute pain problems, they could be used as potent alternative remedies in managing chronic persistent pain with minimal side effects.

## 1. Introduction

Pain is a typical symptom that is frequently linked to neurological and musculoskeletal disorders [1,2]. It is more prevalent in women and older persons, and there are rising tendencies in general populations [1,2,3]. Pain is also the primary symptom of several common clinical illnesses that are frequently coexisting [1,2]. Chronic pain is common among older persons, causing severe suffering, disability, social isolation, and higher healthcare expenses and burdens [3,4]. Furthermore, chronic pain medication is generally ineffective and limited by adverse effects [3,5].

Nonsteroidal drugs are often recommended as first choice remedies for pain control; however, it is important to consider the long-term use safety issues [4,5]. Herbs and nutritional supplements may offer a safer and often effective alternative to nonsteroidal pharmaceuticals for pain management, particularly for long-term use [6,7,8].

Recently, topical analgesic therapies, where the active compounds are applied via the skin and create therapeutically effective concentrations only at the administration site, have gained attention as an innovative approach due to their sufficient efficacy and comparatively fewer systemic side effects and drug–drug interactions [9,10]. In this work we overview the main natural herbal painkillers, discuss their efficacy and safety, and their prospective use as topical pain relievers.

## 2. Pain Mechanisms and Control

**Figure 1:**
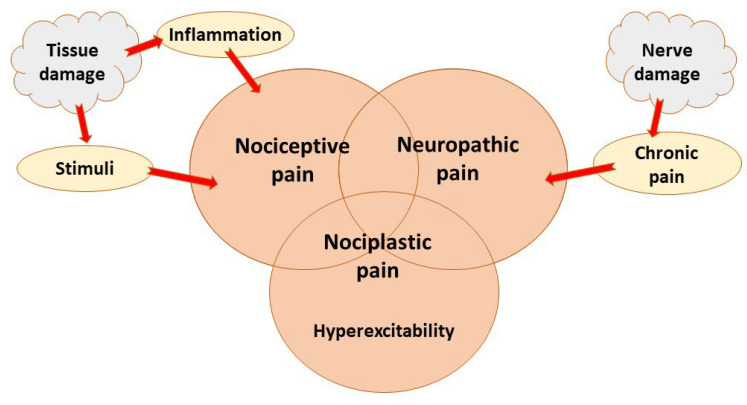
Main pain types.

The definition of pain by the International Association for the Study of Pain (IASP), states “Pain is an unpleasant sensory and emotional experience associated with, or resembling that associated with, actual or potential tissue damage” [2]. There are two main types of pain: nociceptive and neuropathic pain [1,2,3]. Pain that is characterized by both nociceptive and neuropathic properties is called nociplastic [1,2,3] (Figure 1).

The biggest difference between nociceptive and neuropathic pain is that nociceptive pain is caused by tissue damage [1,2,3]. Nerve cell endings that initiate sensations of pain are nociceptors that can respond to chemical (inflammatory), mechanical, or thermal stimuli [1]. Nociception involves the four processes of transduction, transmission, perception, and modulation [1]. Peripheral nociceptors are sensitized during inflammation [1,2]. The types of ion channels present in a nociceptor influence the excitability and behavior of the cell. The nociceptors transmit the electrical signaling information to the dorsal horn of the spinal cord, where a complex network of neurons process nociception and pain via synaptic connections [1,2]. Not a single pathway is responsible for the perception of pain in the CNS; rather, many pathways are involved in the transmission of pain signals to the cerebral cortex [1,2]. The sense of pain is the outcome of the processing of electrical signals in distinct brain areas. This describes the variety of emotions a person may have when experiencing pain [1,2].

**Figure 2:**
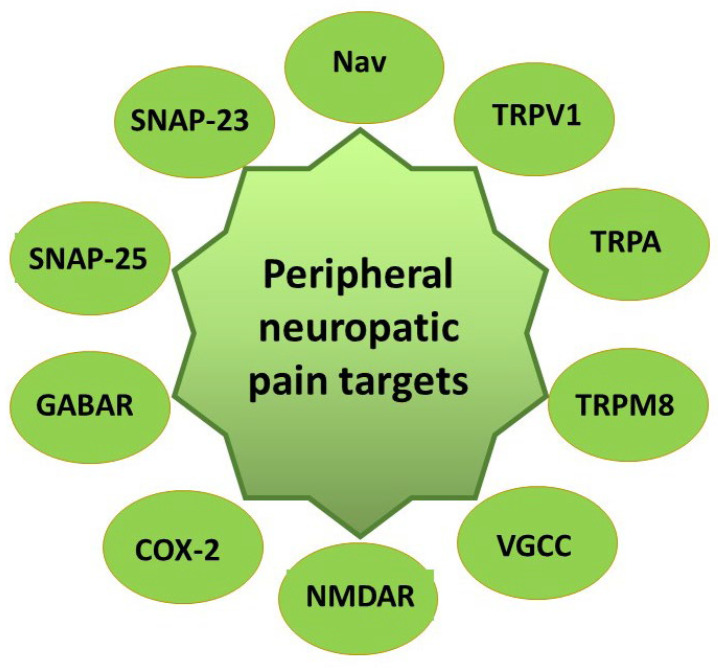
Peripheral neuropathic pain targets (non-opioid). Nav—voltage gated sodium channels, TRPV1—transient receptor potential vanilloid 1 channel, TRPA—transient receptor potential ankyrin channel, TRPM8—transient receptor potential melastatin 8 channels, VGCC—voltage-gated calcium channels, NMDAR—N-methyl-D-aspartic acid receptor, COX-2—cyclooxygenase 2, GABAR—GABA receptor, SNAP-23 and SNAP-25—SNARE protein complex components.

Neuropathic pain is caused by nerve damage and can occur without the presence of noxious stimuli [3]. Much neuropathic pain is chronic [3]. Neuropathic pain is characterized by hyperalgesia and allodynia and is caused by a disease or lesion of the central or peripheral nervous system [11]. Recent evidence suggests that pro-inflammatory cytokines, such as interleukin-1b (IL-1b), released by immune cells, microglia, and astroglia in the spinal cord play crucial roles in the etiology of neuropathic pain [12]. These agents can trigger a cascade of neuroinflammation-related events that may prolong and aggravate the initial insult, ultimately resulting in pain and chronicity [13]. Furthermore, inflammation increases the expression of cyclooxygenase-2 (COX-2) and results in the production of prostaglandins (PGE) [14]. PGE2 is a factor that causes pain. It can sensitize primary sensory neurons, induce central sensitization, and promote the release of pain-related neuropeptides [15]. Metalloproteinases (MMPs) are predominantly associated with tissue remodeling and inflammation in neurodegenerative diseases [16]. In the chronic phase of neuropathic pain, these substances play crucial roles in nociception and hyperalgesia [17,18] (Figure 2).

Based on the evidence from randomized clinical trials, the best options to alleviate local neuropathic pain are topical analgesics including 5% lidocaine patches, 8% capsaicin patches, and botulinum toxin A [10].

Lidocaine is a local anesthetic aminoamide acting mainly via blockage of voltage-gated sodium channels (Nav). It binds preferably to the open or inactivated Nav, suppressing intracellular influx of Na^+^, thus inhibiting the electrical impulse initiation and propagation. Phenytoin, a member of the hydantoin family, acts via a non-selective blockade of Nav, thus resulting in decreased neuron firing and exerting anticonvulsant and anti-neuropathic activities [19]. Ambroxol is a mucolytic and potent local anesthetic drug acting via Nav inhibition [20] and proinflammatory cytokine suppression, [21] reducing repetitive firing and neuronal excitability mainly due to Nav1.8 subtype blockage [20]. Amitriptyline, a tricyclic antidepressant, exerts antinociceptive activity due to the inhibition of Nav1.7, Nav1.8, and Nav1.9 subtypes [22]. A tricyclic antidepressant, doxepin, also possesses Nav-blocking properties and can be used topically for the treatment of local neuropathic pain [23]. Other drugs capable of controlling local neuropathic pain via Nav channel inhibition include funapide (a selective Nav1.7 suppressor) [24], NSAIDs, opioids, agonists of α2 adrenergic receptors [25].

Another group of topical local neuropathic pain-controlling drugs belongs to the transient receptor potential channel (TRP) family modulators [10]. The proteins from this channel family are responsible for the development and support of chronic pain [10]. Capsaicin is a selective agonist of transient receptor potential vanilloid 1 channel (TRPV1), activating it and later resulting in desensitization [26]. Cannabinoids have a similar mechanism of action [27]. The NSAIDs diclofenac, xefocam, and ketorolac suppress TRPV1 and transient receptor potential ankyrin channel (TRPA) [28]. Cooling drugs, such as menthol, at lower concentrations act via activation of transient receptor potential melastatin 8 (TRPM8) [29].

Voltage-gated calcium channel modulators are gabapentin, an antiepileptic and anxiolytic drug [30], and, in preclinical models, lidocaine, phenytoin, and menthol [10].

N-methyl-D-aspartic acid receptor modulators suppress the receptors, thus preventing a secondary hyperalgesia [31]. This mechanism is common for ketamine [32], and, at preclinical level, for antidepressants [33], diclofenac [34], and lidocaine [35]. One of the mechanisms of action of NSAID diclofenac is proinflammatory prostaglandin synthesis suppression via cyclooxygenase -2 (COX) inhibition resulting in antinociception [25]. GABA receptor (GABAR) activation by agonists leads to an intracellular increase in K^+^ ions and decrease in Ca^2+^ ions, resulting in the inhibition of signal suppression [36,37]. A selective agonist of GABAR is baclofen [38]. Potent antinociceptive drugs are α-adrenergic receptor modulators, clonidine (α2-AR agonist) [39] and prazosin (α1-AR antagonist) [40]. Exocytotic neurotransmitter release is modulated by SNAP-25 and SNAP-23 proteins, which are targeted by botulinum toxin A [41,42,43].

Thus, medicines used for local neuropathic pain treatment exert their actions via a wide variety of receptors, ion channels, and proteins, opening many possibilities to develop novel topical analgesic formulations. Topical application is an important administration route for drugs requiring local action on the skin, thereby avoiding their systemic absorption and adverse side effects.

## 3. Natural Herbal Analgesic Substances

### 3.1. Lavender (Lavandula angustifolia Mill.)

True lavender, or *Lavandula angustifolia* Mill., is a tiny perennial shrub in the Lamiaceae family common to Mediterranean regions that is highly valued for its decorative qualities and the aromatic and therapeutic characteristics of its essential oils [44]. Lavender (*Lavandula angustifolia* Mill.) has been traditionally used to alleviate neurologic conditions, such as insomnia and anxiety, and as an analgesic remedy [44]. The main active compounds of lavender include linalool, linaloyl acetate, perillyl alcohol, and 1,8 cineole (eucalyptol) [44].

Lavender essential oil consistently inhibited spontaneous nociception and the effect was comparable to that of tramadol in the in vivo model of formalin-induced pain in male Wistar rats [45]. Furthermore, lavender essential oil alleviated neuropathic pain in mice with spared nerve injury after an acute oral administration of 100 mg/kg. The mechanisms of the observed effect were related to the decreased phosphorylation of ERK1, ERK2 and JNK1 kinases, and decreased the levels of iNOS in the spinal cord, as well as the involvement of the endocannabinoid system [46]. The active compound of lavender essential oil, linalool, has been found to be responsible for the reduction of mechanical hyperalgesia in conditions of chronic inflammatory and neuropathic pain via modulation of peripheral and central opioid and cannabinoid 2 receptors [47]. Recent findings showed that olfactory stimulation by lavender essential oil inhibited nociceptive signal processing at the input stage of the central trigeminal system in mice in vivo [48]. Lavender oil was more effective than ibuprofen in stress-related disorders in an in vivo study on rats where exploratory, anxiolytic, and anti-depressant activities were evaluated using open field test, light/dark transition box activity, and forced swim test [49].

In the single-blinded, randomized clinical trial, 90 patients with osteoarthritis of the knee were randomly assigned to three groups: intervention (aromatherapy massage with lavender essential oil), placebo (massage with almond oil), and control (without massage) [50]. The pain was assessed with Visual Analogue Scale immediately after the intervention and after 1 and 4 weeks after it. Based on the pain severity after one week of the intervention, aromatherapy massage with lavender essential oil could relieve pain in patients with knee osteoarthritis [50].

The results of a systematic review of eight studies of aromatherapy massage with lavender essential oil revealed that lavender was effective in alleviating labor pain and anxiety [51]. In six trials involving 415 participants, lavender significantly reduced pain in women with episiotomy assessed with Visual Analog Scale [52]. Short-term (up to two weeks) beneficial effects of lavender essential oil in reducing emotional stress, pain, muscular tension, and fatigue were seen, but no long-lasting effects of aromatherapy for cancer patients have been reported in a systematic review [53]. Ninety patients undergoing hemodialysis with arteriovenous fistula were included in a randomized controlled and experimental clinical trial to evaluate the effects of topically applied and inhaled lavender essential oil on the intensity of pain [54]. Both applications significantly decreased the severity of pain at the time of arterial insertion of needles [54]. The effects of aromatherapy massage with lavender essential oil on neuropathic pain severity and quality of life was evaluated in an open label randomized controlled clinical study of 46 patients [55]. The intervention group received aromatherapy massage three times per week for a period of 4 weeks. Neuropathic pain scores significantly decreased, and quality of life scores significantly improved in the intervention group in the fourth week of the study [55]. A massage application with lavender essential oil had a longer effect in decreasing postoperative pain of patients in the first hours after gynecologic surgery in a randomized, placebo-controlled study of 45 patients where the pain levels of the patients were evaluated with a Verbal Rating Scale) at the 30th min and the 3rd h after the application [56]. Aromatherapy with lavender essential oil helped in control of pain intensity for 172 abdominal surgical patients assessed in a randomized control trial [57]. Aromatherapy massage with lavender oil was effective in the management of painful myogenous temporomandibular disorders and limited mouth opening in a randomized controlled clinical trial of 91 patients [58]. Treatment with lavender aromatherapy reduced opioid demand of morbidly obese patients undergoing laparoscopic adjustable gastric banding in a prospective randomized placebo-controlled study carried out on 54 patients [59].

Lavender is “generally recognized as safe” (GRAS) as a food by the U.S. Food and Drug Administration [44]. Lavender is well tolerated in adults, but no data about the safety and efficacy of lavender are reported for infants and nursing mothers. Lavender oil exerts antiandrogenic and estrogenic activity, therefore topical application around the breast should not be performed [44]. Prolonged use of essential oil could cause the local skin irritation [53].

### 3.2. Rosemary (Rosmarinus officinalis L.)

Rosemary (*Rosmarinus officinalis* L.) belongs to the Lamiaceae family. It is an evergreen, perennial, branched shrub with fragrant needle-shaped dark green leaves that can grow up to one meter high [60]. It is native to the Mediterranean region, and its leaves are used extensively in the Mediterranean diet as spices and flavorings [60]. Rosemary (*Rosmarinus officinalis* L.) has been traditionally used as a mild antispasmodic and mild analgesic agent to alleviate rheumatic pain, spasms, neuralgia, headaches, migraine, and nervous agitation [18]. Its main constituents comprise carnosic acid, carnosol, rosmarinic, and ursolic acids [60].

Rosemary oil was found to have agonistic effects on the α1 and α2 adrenergic receptors at a concentration of up to 25 µL/L in in vitro experiments with circular smooth muscle strips of guinea pig stomach, resulting in improved blood circulation and reduced pain [61]. Rosemary essential oil (100, 300 and 600 mg/kg, IP) demonstrated a dose-dependent antinociceptive effect in vivo, evaluated by the significant decrease in the dysfunction in the pain-induced functional impairment model in the rat [62]. Furthermore, the antinociceptive properties of rosemary ethanolic extract were comparable to those of tramadol (3.16–50 mg/kg, IP in mice, and 1.0–31.62 mg/kg, IP in rats) or acetylsalicylic acid (31.62–562.32 mg/kg, PO) in vivo in a model of arthritic pain in rodents evaluated by acetic acid-induced writhing test and formalin test [63]. Rosemary essential oil (20 mg/kg, PO) was effective in reducing pain in combination with acetaminophen (60 mg/kg, IP) and codeine (30 mg/kg, IP) in vivo in mice [64]. Furthermore, rosemary essential oil (0.1, 0.5, and 1.0% *w*/*w*) could increase percutaneous absorption of diclofenac topical gel in mice [65]. The nano emulsion containing essential oils of peppermint and rosemary reduced osteoarthritis pain by increasing antioxidant capacity and ameliorating the histopathological features of the rats’ knee joint [66]. Hydroalcoholic extract of rosemary (10–50 mg/kg, IP) and carnosol (0.5–2 mg/kg, IP) inhibited formalin-induced pain and inflammation in mice via action on nicotinic receptors [67]. Micromeric, oleanolic, and ursolic acids from rosemary exerted anti-inflammatory and antinociceptive activities in vivo in a model of arthritic pain in mice evaluated by acetic acid-induced writhing test and formalin test with efficacy comparable to a typical clinical analgesic, ketorolac (10 mg/kg, IP) [68].

Non-volatile rosemary compounds, cirsimaritin, rosmanol, and salvigenin (50–200 mg/kg), could modulate GABA receptors and exert CNS activity in vivo in mouse models of antinociception [69].

The topical application of rosemary reduced the frequency and severity of recurrence of musculoskeletal pain in hemodialysis patients [70]. Aromatherapy massage with rosemary essential oil could increase life quality scores and reduce the severity of neuropathic pain in 46 patients with diabetes [55]. Although there are many studies on the antinociceptive effect of rosemary in vitro and in vivo, more studies should be performed to evaluate its safety and efficacy in clinical practice.

Rosemary is classified as “generally safe” or GRAS by the U.S. Food and Drug Administration, however, high dosage or prolonged rosemary or its active compound administration should be avoided [18]. Higher doses of rosemary may cause miscarriage, and pregnant and nursing women should not take rosemary as a supplement [60,71]. People with high blood pressure, ulcers, Crohn’s disease, or ulcerative colitis should not take rosemary preparations [72].

### 3.3. Peppermint (Mentha piperita L.)

Peppermint (*Mentha piperita* L) is a perennial herb in the Lamiaceae family that is glabrous and intensely fragrant [73]. It is grown in temperate regions of Europe, Asia, the United States of America, India, and Mediterranean countries due to its distinct aroma [73]. Peppermint (*Mentha piperita* L.) and its main active ingredient menthol has been used as an analgesic remedy to alleviate gastric problems, neuromuscular pain, and visceral pain [73]. Peppermint contains menthol, menthone, and menthyl acetate as major ingredients, and 1,8-cineole, pulegone, caffeic acid, flavonoids, tannins, and bitter substances as minor ingredients [73]. Menthol induces the sensation of cooling by activating TRPM8, an ion channel in cold-sensitive peripheral sensory neurons, and could also modulate neurotransmitter receptors, the irritant receptor, TRPA1, and voltage-gated ion channels [74]. Peppermint oil also exerts smooth muscle relaxant properties [75]. Both peppermint oil and its active compound menthol could block calcium channels (IC50 7.7–28.1 µg/mL) in guinea pig ileal smooth muscles in vitro [76]. Furthermore, menthol (0.1–30 mM) enhanced circular smooth muscle relaxation, directly inhibiting gastrointestinal smooth muscle contractility via the inhibition of Ca^2+^ influx through sarcolemma L-type Ca^2+^ channels [77]. This activity was not related to the transient receptor potential cation channel subfamily M member 8 (TRPM8) activation or signaling via nitrous oxide [77]. Peppermint oil could also regulate the enteric nervous system via modulation of the transient receptor potential cation channel, subfamily A, member 1 (TRPA1) receptor, external Ca^2+^ release, and activation of G-protein [78]. Peppermint oil could decrease visceral pain via modulation of gut-located TRPM8 and/or TRPA1 receptors of the transient receptor potential cation channel superfamily [74,75,79].

Twelve randomized trials were overviewed in a meta-analysis on the use of the peppermint oil in the treatment of irritable bowel syndrome, with results indicating that peppermint oil is a safe and effective therapy for pain and global symptoms [80]. Peppermint oil could also alleviate pain in pediatric functional abdominal pain disorders [81], functional dyspepsia [82], and reduce colonic spasm in colonoscopy [83]. Peppermint oil, due to its smooth muscle relaxation properties, could also alleviate pain in a pilot study of 38 patients suffering from dysphagia and chest pain [84]. In a randomized controlled trial of 80 cardiac patients subjected to intravenous catheterization, aromatherapy (inhalation of peppermint essence) could significantly reduce pain and anxiety [85].

Cutaneous application of a menthol 10% solution could exert significant pain relief, as evaluated by the visual analog scale in a randomized, double-blind, placebo-controlled, crossed-over study of 35 patients with 118 migraine (without aura) attacks [86]. Nasal application of peppermint essential oil (1.5%) caused considerable reduction in the intensity and frequency of headache and decreased pain similar to lidocaine (4%) in a double-blind, parallel, randomized controlled trial of 120 adult patients with a diagnosis of migraine [87]. Menthol was also effective in the case of a tension-type headache [88]. Cutaneous application of peppermint oil (containing 10% menthol) effectively decreased neuropathic pain in a patient with postherpetic neuralgia [89].

In a randomized clinical trial of 55 breastfeeding women, menthol essence (four drops on the nipple and areola after each feeding for 10 or 14 days) significantly reduced nipple fissure pain, as evaluated by the visual analog scale [90]. In a double-blind randomized controlled trial of 126 breastfeeding mothers, lanolin, peppermint, and dexpanthenol creams equally effectively reduced the pain of traumatic nipples [91].

Peppermint is “generally recognized as safe” (GRAS) as a food by the U.S. Food and Drug Administration, however, large doses can cause heartburn, nausea, and vomiting [73]. Allergic reactions, including headache, have been reported to menthol [73]. If peppermint is used on the nipples, it should be used after nursing and wiped off before the next nursing [90]. Peppermint is not recommended for patients who have a hiatus hernia, gastroesophageal reflux, arrhythmia, or hemolytic anemia [73].

### 3.4. Ginger (Zingiber officinale Roscoe)

Ginger, the rizhome of *Zingiber officinale* Roscoe (Zingiberaceae), is a plant originating from in the Indo-Malayan region, and is cultivated mainly in the tropics of Asia, Africa, America, and Australia [92,93,94]. It has been used as a spice and medicine for over 2000 years, and more recently as dietary supplement [92,93,94]. Ginger (*Zingiber officinale* Roscoe) exerts a wide range of biological activities, including pain relief, protection against nausea, vomiting, male infertility, alleviating diabetes, reducing inflammation, and fighting obesity [92,93,94]. The main active compounds of ginger are terpenes and phenolics, especially gingerols [94]. The anti-inflammatory action of ginger was thought to be related to the inhibition of key enzymes of the arachidonate metabolic pathway: cyclooxygenase (COX) and 5-lipoxygenase (LOX) [95]. Furthermore, it could also suppress the induction of inflammatory genes [96].

The ginger active compound zerumbone inhibited IL-1β, IL-6, and TNF-α in a mouse model of neuropathic pain, thus demonstrating hyperalgesic and antiallodynic activities [97]. In a rat model of oral ulcerative mucositis, ginger active compounds [6]-gingerol and [6]-shogaol reduced pain by acting on sodium channels [98]. Ginger oil (for one month PO) significantly suppressed acute carrageenan-, dextran-, and formalin-induced chronic inflammation, as well as acetic acid-induced writhing movements in mice [99].

Ginger (250 mg PO) effectively decreased postpartum pain severity in a double-blinded, randomized, placebo-controlled trial of 128 mothers [100]. Ginger (250 mg PO) was as effective as mefenamic acid (250 mg) and ibuprofen (400 mg) in relieving pain in women with primary dysmenorrhea in a double-blind comparative clinical trial conducted on 150 participants [101]. The effects of ginger in alleviating the symptoms of primary dysmenorrhea were overviewed in a systematic review and meta-analysis of randomized clinical trials, and the results confirmed the efficacy of this therapy assessed by a pain visual analogue score [102].

Sublingual feverfew/ginger effects were evaluated in a double-blind placebo-controlled pilot study of 60 patients with 221 migraine attacks [103]. The results showed that sublingual feverfew/ginger is a safe and effective therapy to alleviate migraine symptoms [103].

In a case study, a patient with osteoarthritis received 7 consecutive days of ginger therapy in an integrative medical center and afterwards for a further 24 weeks at home [104]. Over the course of 24 weeks, ginger therapy significantly reduced osteoarthritis symptoms, with no side effects being recorded [104]. Furthermore, ginger not only decreased rheumatoid arthritis symptoms, but could also stop rheumatoid arthritis-induced bone destruction [105].

Ginger has a long history of use as a food and medicine and is “generally recognized as safe” (GRAS) as a food flavoring by the U.S. Food and Drug Administration [93]. Mild common side effects are stomach upset, nausea, eructation, and dyspepsia [93]. Due to antiplatelet activity, ginger should be administered with precautions for patients using anticoagulative drugs [93].

### 3.5. Feverfew (Tanacetum parthenium L.)

*Tanacetum parthenium* (L.) Schultz-Bip is commonly known as feverfew (Family: Asteraceae). This perennial herb ranges in height from 14 to 45 cm and has feather-like, greenish-yellow leaves [106,107]. It is widespread in Europe’s hedgerows and wastelands and has been grown as a decorative and therapeutic plant for centuries [106,107]. Historically, feverfew leaves or their infusion have been used to alleviate symptoms associated with a wide range of medical conditions, including but not limited to: fevers, allergies, migraine headaches, rheumatoid arthritis, tooth pain, nausea, stomach pain, infertility, menstruation, and labor problems [106,107]. The main active compounds of the feverfew are sesquiterpene lactones, especially parthenolide, flavonoid glycosides, and pinenes [106,107].

Feverfew extract (10, 20, 40 mg/kg, PO) significantly reduced acetic acid-induced writhing in mice and carrageenan-induced paw edema in rats, demonstrating antinociceptive and anti-inflammatory properties [108]. Feverfew extract was also effective in relieving painful diabetic peripheral neuropathy in streptozotocin-diabetic rats [109]. The multiple effects of feverfew flower extract were evaluated after acute administration of 30–1000 mg/kg PO in mice [106]. The writhing test was used to determine analgesic efficacy. There was a dose-dependent decrease in the frequency of abdominal contractions when the floral extract was administered [106]. Feverfew extract reduced mechanical hypersensitivity related to the acute inflammatory phase induced by carrageenan similarly to diclofenac and ibuprofen, as assessed by paw pressure test [106]. An increase in pain threshold, which peaked 30 min after treatment, was observed in an osteoarthritis model induced by intraarticular injection of monoiodoacetate [106]. Moreover, feverfew extract was effective in a chronic constriction injury model of neuropathic pain, showing activity similar to the antiepileptic drug gabapentin [106]. Feverfew flower extract was found to dramatically diminish mechanical hypersensitivity generated by multiple doses of the anticancer drug oxaliplatin and antiviral drug dideoxycytidine, demonstrating efficacy in chemotherapy-induced neuropathic pain [106].

Patients using feverfew for up to 6 months of treatment reported fewer headaches, according to a study involving eight individuals who received feverfew medication and nine placebo-controlled patients [110]. These findings were confirmed in a randomized double-blind placebo-controlled trial including 72 migraine patients [111]. A systematic review of randomized controlled trials and their clinical findings and potential implications suggested a benefit of feverfew in migraine prophylaxis [112].

A daily dosage of 125 mg of a dried feverfew leaf preparation containing at least parthenolide 0.2% is recommended for the prevention of migraine, the effect being similar to a known 5-HT antagonist, methysergide maleate [107].

Feverfew is generally considered as safe. Mouth ulceration and nervous system reactions have been reported as adverse reactions when taking feverfew preparations, although they were mild and did not lead to discontinuation [107] Side effects from feverfew can include abdominal pain, indigestion, gas, diarrhea, nausea, vomiting, and nervousness. Rarely, allergic reactions to feverfew have been reported [107]. People who are allergic to chamomile, ragweed, or yarrow may also be allergic to feverfew and should therefore avoid taking it. Feverfew may increase the risk of bleeding, especially taken together with blood-thinning medications, and may interact with anesthesia [107]. Pregnant and nursing women, as well as children under 2, should not take feverfew. Treatment with feverfew lasting for more than one week should not be discontinued abruptly, as it might result in rebound headache, anxiety, weariness, muscular stiffness, and joint discomfort [107].

### 3.6. Turmeric (Curcuma longa L.)

*Curcuma longa* L., or Indian saffron, is a herbaceous, perennial, rhizomatous plant of the Zingiberaceae family, native to south-eastern Asia and widely used as a spice especially in Indian, Middle Eastern, Thai, and other Asian cuisines [113]. Traditional uses of turmeric (*Curcuma longa* L.) include alleviation of inflammation, better wound healing, and use as an antioxidant, painkiller, and antibacterial compound [113]. Curcumin, a polyphenolic molecule, is responsible for turmeric biological activities [113]. Curcumin can regulate inflammatory cytokines such as interleukin (IL)-1 beta, IL-6, IL-12, Tumor necrosis factor (TNF)-alpha, interferon (IFN) gamma, and associated AP-1, NF-kappa B, and JAK-STAT signaling pathways [113]. Due to the ability to suppress inflammation, it has been used in autoimmune diseases such as rheumatoid arthritis, inflammatory bowel disease, and multiple sclerosis [113].

In preclinical studies, it was demonstrated that curcumin can reduce inflammatory and neuropathic pain [113,114]. Through its antioxidant and anti-inflammatory properties, curcumin can alleviate inflammatory bowel diseases [115]. Curcumin (100 mg/kg, 5 days, PO) suppressed the amount of NF-ĸB, COX-2, 5-LOX, and iNOS expression in inflammatory bowel disease, and inhibited toll-like receptor-4 (TLR-4)-induced NF-ĸB activation in experimental colitis in Sprague–Dawley male rats [116]. Curcumin (60, 120 mg/kg, 7 days, IP) reduced the production of spinal IL-1β through inhibition of the aggregation of NAcht leucine-rich repeat protein 1 inflammasome and activation of the Janus kinase 2-signal transducer and activator of transcription 3 signaling pathway in astrocytes, and alleviated neuropathic pain [29].

In a double-blind, randomized, placebo-controlled trial involving 160 patients with knee osteoarthritis, turmeric extract (4 months, PO) decreased pain as assessed by visual analog scale, and reduced the presence of inflammatory markers in patients’ blood [117]. The efficacy of curcumin (500 mg capsule, 3 × day PO for 28 days) and diclofenac (50 mg tablet 2 × day PO for 28 days) to alleviate pain was compared in a randomized, open-label, parallel, active controlled clinical trial involving 139 patients with knee osteoarthritis [118]. The severity of pain was evaluated by visual analogue scale score at days 14 and 28. Curcumin had a similar efficacy compared to diclofenac, but demonstrated better tolerance among patients with knee osteoarthritis [118]. Furthermore, in a double-blind randomized placebo-controlled trial involving 72 older adults with osteoarthritis-related knee pain, curcumin 5% ointment (2 × day, 6 weeks) significantly decreased the mean pain intensity [119].

Turmeric is generally safe and well tolerated. Higher doses are needed to produce a systemic effect because of its limited absorption [113,114]. Curcumin can inhibit platelet-activating factor and arachidonic acid platelet aggregation; therefore, concomitant use of turmeric with other drugs with similar pharmacologic potential, such as naproxen, may increase the risk of bleeding, and therapy modification is recommended [113,114].

## 4. Emerging Herbal Therapies

### 4.1. Clove (Syzygium aromaticum L.)

Clove (*Syzygium aromaticum*) is an aromatic evergreen plant that belongs to the family of Myrtaceae [120,121]. It is an aromatic plant widely cultivated in tropical and subtropical countries, rich in volatile substances. Clove is one of the most valuable spices and has been used for centuries as a food preservative and for medicinal purposes [120,121].

Eugenol, the primary active component of clove (*Syzygium aromaticum* L.) oil, has been used in traditional medicine to alleviate dental pain [120,121]. Clove’s spicy aroma and pungent flavor are attributed to eugenol, a volatile bioactive naturally occurring phenolic monoterpenoid that is a member of the phenylpropanoids [120,121]. Similar to the capsicum in peppers, clove oil gives the skin or mucosa a warming feeling following application [120,121]. The transient receptor potential cation channel subfamily V member 1 (TRPV-1), also known as vanilloid receptor 1, is responsible for clove oil’s therapeutic effects. The receptor desensitizes nerve endings close to the skin’s surface when it is activated [120,121]. Additionally, clove oil contains potent antibacterial qualities that can aid in wound healing and infection prevention [120,121]. Although clove oil is used traditionally, detailed clinical investigations should be performed before its wider application as an alternative remedy for pain control.

If used properly, clove oil is regarded as safe, however, excessive or repeated use can make it toxic [120,121]. The most frequent adverse effect of clove oil is tissue irritation, which manifests as discomfort, swelling, redness, and a burning (as opposed to warming) sensation [120,121].

### 4.2. Clover (Trifolium Species)

Clover (genus *Trifolium*) is a family of roughly 300 annual and perennial plants (Fabaceae) [122]. Clovers are often short-lived herbs with alternate complex leaves that are typically composed of three toothed leaflets [122]. Except for Southeast Asia and Australia, clover grows in most temperate and subtropical regions of the world; cultivated species have naturalized in temperate regions worldwide [122]. The plants can be cultivated as a cover crop or utilized as a green manure, in addition to being helpful as livestock feed. Clover honey is a frequent secondary product of clover production, as the blossoms are particularly attractive to bees [122].

Clover (*Trifolium*) species in folk medicine are used for their analgesic and antiseptic properties, and as a remedy to alleviate rheumatic disturbances [123]. The main bioactive compounds in clover species are estrogenic isoflavones: daidzein, genistein, formononetin, biochanin A, coumestrol, and naringenin. Minor compounds include other flavonoids, pterocarpans, coumarins, and tyramine [122].

Red clover is mainly used as a phytoestrogen for the treatment of menopause symptoms and bone and cardiovascular health support during pre-menopause or menopause [124,125,126,127].

Although there are no clinical data available yet, in vivo studies show that in the case when pain threshold was reduced due to estrogen deprivation, red clover extract (500 mg/kg of body weight) given to ovariectomized (OVX) and normal (control) rats for 90 and 180 days could restore it to normal levels, as assessed by tail flicking and formalin test methods [128]. Moreover, the clover active compound formononetin exerted anxiolytic activity in a chronic pain model in mice in vivo via the suppression of inflammation and neuronal hyperexcitability [129]. However, detailed clinical studies are needed to evaluate clover use in folk medicine for analgesic and antirheumatic purposes.

Clover does not have serious side effects during prolonged use [123,124,130]. Clover preparations may include headache, nausea, and rash [123,124,130]. Pregnant or breastfeeding women should not take red clover [123,124,130].

## 5. Pros and Cons of Natural Painkillers: Their Efficacy vs. Traditional Therapy

Pain, a global public health priority, is an unpleasant sensory and emotional experience related to real or potential tissue damage [5]. About twenty percent of adults globally experience pain, and ten percent are newly diagnosed with chronic pain each year [4]. Current pain management strategies rely mostly on nonsteroidal anti-inflammatory drugs (NSAIDs) [5]. These medications are the first-line treatment for mild to moderate pain, followed, if ineffective, by stronger opioids [131]. However, the use of opioids is compromised due to their many negative side effects and possibility of addiction [131]. Opioids are capable of causing drowsiness, nausea/vomiting, constipation, tolerance, physical dependency, and respiratory depression [131].

**Figure 3:**
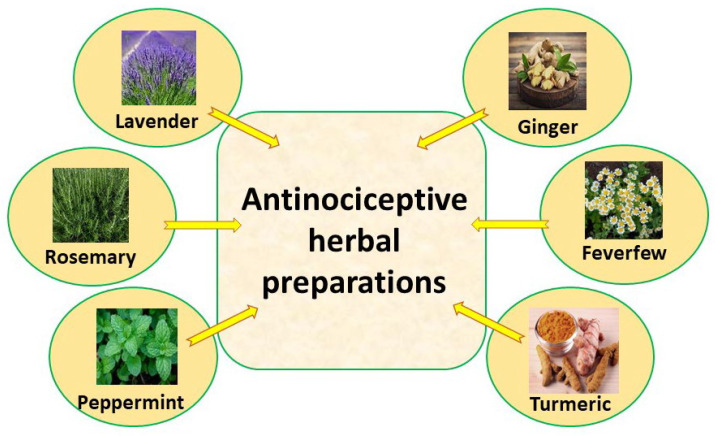
Main herbs exerting antinociceptive activity.

Therefore, it is necessary to develop alternatives for pain management, particularly those derived from plants [6,7] (Figure 3).

**Figure 4:**
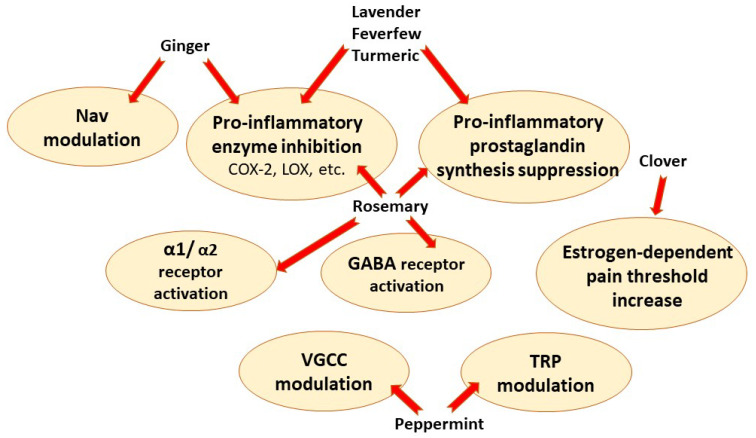
Main analgesic mechanisms of herbal painkillers. Nav—voltage gated sodium channels, TRP–transient receptor potential channels, VGCC—voltage-gated calcium channels, COX-2—cyclooxygenase 2, LOX—5-lipoxygenase.

Plant extracts, instead of simple components, allow for synergistic properties or efficacy against multiple targets. Phenolic plant compounds are strong antioxidants with potent anti-inflammatory properties; thus, suppression of inflammation signaling pathways is one of the main mechanisms of action of plant-derived painkillers. Furthermore, different plant extracts also have their specific targets, such as GABA receptors and transient receptor potential channels, summarized in Figure 4.

Chronic pain patients frequently utilize a variety of complementary therapies. Increasingly, these treatments have been subjected to the same rigorous evaluation as all modern practices based on scientific evidence [1]. More than 60% of patients with chronic pain sooner or later chose alternative treatments to drugs [6].

The main question about using herbal remedies to alleviate acute or chronic pain is related to the ability to respond quickly. Traditional therapies with NSAIDs or analgesic drugs are fast enough and very effective, however, the use of narcotic drugs is always related to heavy and hardly tolerable side effects [1]. In such a situation, herbal-based drugs might have anti-inflammatory, analgesic, and muscular relaxant characteristics with fewer side effects [7]. The mechanism of action of a number of herbs has already been identified, and is similar to that of synthetic drugs [7]. However, there are herbal remedies where traditional use has demonstrated their efficacy, but the mechanism of action is still unknown [7]. Furthermore, herbs usually consist of ingredient mixtures, while traditional drugs are pure compounds [6,7,8]. Usually, pain-relieving properties of natural herbal drugs originate from their numerous constituents, capable of acting via distinct molecular pathways. However, multiple herbal ingredients could also be responsible for the interaction with other synthetic drugs used by the patient.

Thus, to sum up, plant-derived drugs could be good candidates with negligible side effects for chronic persistent pain management, but not suitable to provide fast relief under acute pain conditions.

## Data Availability

Not applicable.

## References

[1] Basbaum A.I., Bautista D.M., Scherrer G., Julius D. (2009). Cellular and molecular mechanisms of pain. Cell.

[2] Fernandes V., Sharma D., Vaidya S., PA S., Guan Y., Kalia K., Tiwari V. (2018). Cellular and molecular mechanisms driving neuropathic pain: Recent advancements and challenges. Expert Opin. Ther. Targets.

[3] Finnerup N.B., Kuner R., Jensen T.S. (2021). Neuropathic Pain: From Mechanisms to Treatment. Physiol. Rev..

[4] Luchting B., Azad S.C. (2019). Pain therapy for the elderly patient: Is opioid-free an option?. Curr. Opin. Anaesthesiol..

[5] Raymond T.J., Tobin K.A., Rogers T.S. (2021). Nonopioid Pharmacologic Treatments for Chronic Pain. Am. Fam. Physician.

[6] Boyd A., Bleakley C., Hurley D.A., Gill C., Hannon-Fletcher M., Bell P., McDonough S. (2019). Herbal medicinal products or preparations for neuropathic pain. Cochrane. Database Syst. Rev..

[7] Jahromi B., Pirvulescu I., Candido K.D., Knezevic N.N. (2021). Herbal Medicine for Pain Management: Efficacy and Drug Interactions. Pharmaceutics.

[8] Casale R., Symeonidou Z., Ferfeli S., Micheli F., Scarsella P., Paladini A. (2021). Food for Special Medical Purposes and Nutraceuticals for Pain: A Narrative Review. Pain Ther..

[9] Casale R., Symeonidou Z., Bartolo M. (2017). Topical Treatments for Localized Neuropathic Pain. Curr. Pain Headache Rep..

[10] Kocot-Kępska M., Zajączkowska R., Mika J., Kopsky D.J., Wordliczek J., Dobrogowski J., Przeklasa-Muszyńska A. (2021). Topical Treatments and Their Molecular/Cellular Mechanisms in Patients with Peripheral Neuropathic Pain-Narrative Review. Pharmaceutics.

[11] Treede R.D., Jensen T.S., Campbell J.N., Cruccu G., Dostrovsky J.O., Griffin J.W., Hansson P., Hughes R., Nurmikko T., Serra J. (2008). Neuropathic pain: Redefinition and a grading system for clinical and research purposes. Neurology.

[12] Sacerdote P., Franchi S., Moretti S., Castelli M., Procacci P., Magnaghi V., Panerai A.E. (2013). Cytokine modulation is necessary for efficacious treatment of experimental neuropathic pain. J. Neuroimmune Pharmacol..

[13] Valsecchi A.E., Franchi S., Panerai A.E., Rossi A., Sacerdote P., Colleoni M. (2011). The soy isoflavone genistein reverses oxidative and inflammatory state, neuropathic pain, neurotrophic and vasculature deficits in diabetes mouse model. Eur. J. Pharmacol..

[14] Gao Y.J., Ji R.R. (2010). Targeting astrocyte signaling for chronic pain. Neurotherapeutics.

[15] Hingtgen C.M., Waite K.J., Vasko M.R. (1995). Prostaglandins facilitate peptide release from rat sensory neurons by activating the adenosine 3’,5’-cyclic monophosphate transduction cascade. J. NeuroSci..

[16] Page-McCaw A., Ewald A.J., Werb Z. (2007). Matrix metalloproteinases and the regulation of tissue remodelling. Nat. Rev. Mol. Cell Biol..

[17] Ji R.R., Xu Z.Z., Wang X., Lo E.H. (2009). Matrix metalloprotease regulation of neuropathic pain. Trends Pharmacol. Sci..

[18] Rahbardar M.G., Amin B., Mehri S., Mirnajafi-Zadeh S.J., Hosseinzadeh H. (2018). Rosmarinic acid attenuates development and existing pain in a rat model of neuropathic pain: An evidence of anti-oxidative and anti-inflammatory effects. Phytomedicine.

[19] Thorn C.F., Whirl-Carrillo M., Leeder J.S., Klein T.E., Altman R.B. (2012). PharmGKB summary: Phenytoin pathway. Pharma. Genom..

[20] Zhu W., Li T., Silva J.R., Chen J. (2020). Conservation and divergence in NaChBac and Na(V)1.7 pharmacology reveals novel drug interaction mechanisms. Sci. Rep..

[21] Sunkari S., Thatikonda S., Pooladanda V., Challa V.S., Godugu C. (2019). Protective effects of ambroxol in psoriasis like skin inflammation: Exploration of possible mechanisms. Int. Immunopharmacol..

[22] Khan M.A., Gerner P., Kuo Wang G. (2002). Amitriptyline for prolonged cutaneous analgesia in the rat. Anesthesiology.

[23] Dworsky Z.D., Bennett R., Kim J.M., Kuo D.J. (2017). Severe medication-induced peripheral neuropathy treated with topical doxepin cream in a paediatric patient with leukaemia. BMJ Case Rep..

[24] Price N., Namdari R., Neville J., Proctor K.J., Kaber S., Vest J., Fetell M., Malamut R., Sherrington R.P., Pimstone S.N. (2017). Safety and Efficacy of a Topical Sodium Channel Inhibitor (TV-45070) in Patients With Postherpetic Neuralgia (PHN): A Randomized, Controlled, Proof-of-Concept, Crossover Study, With a Subgroup Analysis of the Nav1.7 R1150W Genotype. Clin. J. Pain.

[25] Kumamoto E. (2020). Inhibition of Fast Nerve Conduction Produced by Analgesics and Analgesic Adjuvants-Possible Involvement in Pain Alleviation. Pharmaceuticals.

[26] Sharma S.K., Vij A.S., Sharma M. (2013). Mechanisms and clinical uses of capsaicin. Eur. J. Pharmacol..

[27] Starowicz K., Nigam S., Di Marzo V. (2007). Biochemistry and pharmacology of endovanilloids. Pharmacol. Ther..

[28] Nozadze I., Tsiklauri N., Gurtskaia G., Tsagareli M.G. (2016). NSAIDs attenuate hyperalgesia induced by TRP channel activation. Data Brief.

[29] Liu S., Li Q., Zhang M.T., Mao-Ying Q.L., Hu L.Y., Wu G.C., Mi W.L., Wang Y.Q. (2016). Curcumin ameliorates neuropathic pain by down-regulating spinal IL-1β via suppressing astroglial NALP1 inflammasome and JAK2-STAT3 signalling. Sci. Rep..

[30] Bannister K., Qu C., Navratilova E., Oyarzo J., Xie J.Y., King T., Dickenson A.H., Porreca F. (2017). Multiple sites and actions of gabapentin-induced relief of ongoing experimental neuropathic pain. Pain.

[31] Warncke T., Jørum E., Stubhaug A. (1997). Local treatment with the *N*-methyl-D-aspartate receptor antagonist ketamine, inhibit development of secondary hyperalgesia in man by a peripheral action. NeuroSci. Lett..

[32] Kopsky D.J., Keppel Hesselink J.M., Bhaskar A., Hariton G., Romanenko V., Casale R. (2015). Analgesic effects of topical ketamine. Minerva Anestesiol..

[33] Barygin O.I., Nagaeva E.I., Tikhonov D.B., Belinskaya D.A., Vanchakova N.P., Shestakova N.N. (2017). Inhibition of the NMDA and AMPA receptor channels by antidepressants and antipsychotics. Brain Res..

[34] Dong X.D., Svensson P., Cairns B.E. (2009). The analgesic action of topical diclofenac may be mediated through peripheral NMDA receptor antagonism. Pain.

[35] Sugimoto M., Uchida I., Mashimo T. (2003). Local anaesthetics have different mechanisms and sites of action at the recombinant N-methyl-D-aspartate (NMDA) receptors. Br. J. Pharmacol..

[36] Magnaghi V., Ballabio M., Consoli A., Lambert J.J., Roglio I., Melcangi R.C. (2006). GABA receptor-mediated effects in the peripheral nervous system: A cross-interaction with neuroactive steroids. J. Mol. NeuroSci..

[37] Whitehead R.A., Puil E., Ries C.R., Schwarz S.K., Wall R.A., Cooke J.E., Putrenko I., Sallam N.A., MacLeod B.A. (2012). GABA(B) receptor-mediated selective peripheral analgesia by the non-proteinogenic amino acid, isovaline. Neuroscience.

[38] Wu C., Qin X., Du H., Li N., Ren W., Peng Y. (2017). The immunological function of GABAergic system. Front. BioSci..

[39] Dogrul A., Uzbay T.I. (2004). Topical clonidine antinociception. Pain.

[40] Drummond P.D. (2014). Neuronal changes resulting in up-regulation of alpha-1 adrenoceptors after peripheral nerve injury. Neural. Regen. Res..

[41] Cui M., Khanijou S., Rubino J., Aoki K.R. (2004). Subcutaneous administration of botulinum toxin A reduces formalin-induced pain. Pain.

[42] Meng J., Wang J., Lawrence G., Dolly J.O. (2007). Synaptobrevin I mediates exocytosis of CGRP from sensory neurons and inhibition by botulinum toxins reflects their anti-nociceptive potential. J. Cell Sci..

[43] Welch M.J., Purkiss J.R., Foster K.A. (2000). Sensitivity of embryonic rat dorsal root ganglia neurons to Clostridium botulinum neurotoxins. Toxicon.

[44] (2006). Lavender. Drugs and Lactation Database (LactMed).

[45] Silva G.L., Luft C., Lunardelli A., Amaral R.H., Melo D.A., Donadio M.V., Nunes F.B., de Azambuja M.S., Santana J.C., Moraes C.M. (2015). Antioxidant, analgesic and anti-inflammatory effects of lavender essential oil. Ann. Acad. Bras. Cienc..

[46] Sanna M.D., Les F., Lopez V., Galeotti N. (2019). Lavender (*Lavandula angustifolia* Mill.) Essential Oil Alleviates Neuropathic Pain in Mice With Spared Nerve Injury. Front. Pharmacol..

[47] Donatello N.N., Emer A.A., Salm D.C., Ludtke D.D., Bordignon S., Ferreira J.K., Salgado A.S.I., Venzke D., Bretanha L.C., Micke G.A. (2020). Lavandula angustifolia essential oil inhalation reduces mechanical hyperalgesia in a model of inflammatory and neuropathic pain: The involvement of opioid and cannabinoid receptors. J. Neuroimmunol..

[48] Papotto N., Reithofer S., Baumert K., Carr R., Möhrlen F., Frings S. (2021). Olfactory stimulation Inhibits Nociceptive Signal Processing at the Input Stage of the Central Trigeminal System. Neuroscience.

[49] Qadeer S., Emad S., Perveen T., Yousuf S., Sheikh S., Sarfaraz Y., Sadaf S., Haider S. (2018). Role of ibuprofen and lavender oil to alter the stress induced psychological disorders: A comparative study. Pak. J. Pharm. Sci..

[50] Nasiri A., Mahmodi M.A., Nobakht Z. (2016). Effect of aromatherapy massage with lavender essential oil on pain in patients with osteoarthritis of the knee: A randomized controlled clinical trial. Complement. Ther. Clin. Pract..

[51] Tabatabaeichehr M., Mortazavi H. (2020). The Effectiveness of Aromatherapy in the Management of Labor Pain and Anxiety: A Systematic Review. Ethiop. J. Health Sci..

[52] Abedian S., Abedi P., Jahanfar S., Iravani M., Zahedian M. (2020). The effect of Lavender on pain and healing of episiotomy: A systematic review. Complement. Ther. Med..

[53] Boehm K., Büssing A., Ostermann T. (2012). Aromatherapy as an adjuvant treatment in cancer care--a descriptive systematic review. Afr. J. Tradit. Complement. Altern. Med..

[54] Tüzün Özdemir S., Akyol A. (2021). Effect of inhaler and topical lavender oil on pain management of arteriovenous fistula cannulation. J. Vasc. Access.

[55] Gok Metin Z., Arikan Donmez A., Izgu N., Ozdemir L., Arslan I.E. (2017). Aromatherapy Massage for Neuropathic Pain and Quality of Life in Diabetic Patients. J. Nurs. Scholarsh..

[56] Mizrak Sahin B., Culha I., Gursoy E., Yalcin O.T. (2021). Effect of Massage With Lavender Oil on Postoperative Pain Level of Patients Who Underwent Gynecologic Surgery: A Randomized, Placebo-Controlled Study. Holist Nurs. Pract..

[57] Stallings Welden L.M., Leatherland P., Schitter M.B., Givens A., Stallings J.D. (2021). Abdominal Surgical Patients Randomized to Aromatherapy for Pain Management. J. Perianesth. Nurs..

[58] Benli M., Olson J., Huck O., Özcan M. (2020). A novel treatment modality for myogenous temporomandibular disorders using aromatherapy massage with lavender oil: A randomized controlled clinical trial. Cranio.

[59] Kim J.T., Ren C.J., Fielding G.A., Pitti A., Kasumi T., Wajda M., Lebovits A., Bekker A. (2007). Treatment with lavender aromatherapy in the post-anesthesia care unit reduces opioid requirements of morbidly obese patients undergoing laparoscopic adjustable gastric banding. Obes. Surg..

[60] de Oliveira J.R., Camargo S.E.A., de Oliveira L.D. (2019). *Rosmarinus officinalis* L. (rosemary) as therapeutic and prophylactic agent. J. Biomed. Sci..

[61] Sagorchev P., Lukanov J., Beer A.M. (2010). Investigations into the specific effects of rosemary oil at the receptor level. Phytomedicine.

[62] Martínez A.L., González-Trujano M.E., Pellicer F., López-Muñoz F.J., Navarrete A. (2009). Antinociceptive effect and GC/MS analysis of *Rosmarinus officinalis* L. essential oil from its aerial parts. Planta Med..

[63] González-Trujano M.E., Peña E.I., Martínez A.L., Moreno J., Guevara-Fefer P., Déciga-Campos M., López-Muñoz F.J. (2007). Evaluation of the antinociceptive effect of *Rosmarinus officinalis* L. using three different experimental models in rodents. J. Ethnopharmacol..

[64] Raskovic A., Milanovic I., Pavlovic N., Milijasevic B., Ubavic M., Mikov M. (2015). Analgesic effects of rosemary essential oil and its interactions with codeine and paracetamol in mice. Eur. Rev. Med. Pharmacol. Sci..

[65] Akbari J., Saeedi M., Farzin D., Morteza-Semnani K., Esmaili Z. (2015). Transdermal absorption enhancing effect of the essential oil of *Rosmarinus officinalis* on percutaneous absorption of Na diclofenac from topical gel. Pharm. Biol..

[66] Mohammadifar M., Aarabi M.H., Aghighi F., Kazemi M., Vakili Z., Memarzadeh M.R., Talaei S.A. (2021). Anti-osteoarthritis potential of peppermint and rosemary essential oils in a nanoemulsion form: Behavioral, biochemical, and histopathological evidence. BMC Complement. Med. Ther..

[67] Di Cesare Mannelli L., Micheli L., Maresca M., Cravotto G., Bellumori M., Innocenti M., Mulinacci N., Ghelardini C. (2016). Anti-neuropathic effects of *Rosmarinus officinalis* L. terpenoid fraction: Relevance of nicotinic receptors. Sci. Rep..

[68] Martínez A.L., González-Trujano M.E., Chávez M., Pellicer F. (2012). Antinociceptive effectiveness of triterpenes from rosemary in visceral nociception. J. Ethnopharmacol..

[69] Abdelhalim A., Karim N., Chebib M., Aburjai T., Khan I., Johnston G.A., Hanrahan J. (2015). Antidepressant, Anxiolytic and Antinociceptive Activities of Constituents from *Rosmarinus officinalis*. J. Pharm. Pharm. Sci..

[70] Keshavarzian S., Shahgholian N. (2017). Comparison of the Effect of Topical Application of Rosemary and Menthol for Musculoskeletal Pain in Hemodialysis Patients. Iran. J. Nurs. Midwifery Res..

[71] Andrade J.M., Faustino C., Garcia C., Ladeiras D., Reis C.P., Rijo P. (2018). *Rosmarinus officinalis* L.: An update review of its phytochemistry and biological activity. Future Sci..

[72] Borges R.S., Ortiz B.L.S., Pereira A.C.M., Keita H., Carvalho J.C.T. (2019). *Rosmarinus officinalis* essential oil: A review of its phytochemistry, anti-inflammatory activity, and mechanisms of action involved. J. Ethnopharmacol..

[73] (2006). Peppermint. Drugs and Lactation Database (LactMed).

[74] Liu B., Fan L., Balakrishna S., Sui A., Morris J.B., Jordt S.E. (2013). TRPM8 is the principal mediator of menthol-induced analgesia of acute and inflammatory pain. Pain.

[75] Chumpitazi B.P., Kearns G.L., Shulman R.J. (2018). Review article: The physiological effects and safety of peppermint oil and its efficacy in irritable bowel syndrome and other functional disorders. Aliment. Pharmacol. Ther..

[76] Hawthorn M., Ferrante J., Luchowski E., Rutledge A., Wei X.Y., Triggle D.J. (1988). The actions of peppermint oil and menthol on calcium channel dependent processes in intestinal, neuronal and cardiac preparations. Aliment. Pharmacol. Ther..

[77] Amato A., Liotta R., Mulè F. (2014). Effects of menthol on circular smooth muscle of human colon: Analysis of the mechanism of action. Eur. J. Pharmacol..

[78] Kim H.J., Wie J., So I., Jung M.H., Ha K.T., Kim B.J. (2016). Menthol Modulates Pacemaker Potentials through TRPA1 Channels in Cultured Interstitial Cells of Cajal from Murine Small Intestine. Cell. Physiol. Biochem..

[79] Harrington A.M., Hughes P.A., Martin C.M., Yang J., Castro J., Isaacs N.J., Blackshaw A.L., Brierley S.M. (2011). A novel role for TRPM8 in visceral afferent function. Pain.

[80] Alammar N., Wang L., Saberi B., Nanavati J., Holtmann G., Shinohara R.T., Mullin G.E. (2019). The impact of peppermint oil on the irritable bowel syndrome: A meta-analysis of the pooled clinical data. BMC Complement. Altern. Med..

[81] Korterink J.J., Rutten J.M., Venmans L., Benninga M.A., Tabbers M.M. (2015). Pharmacologic treatment in pediatric functional abdominal pain disorders: A systematic review. J. Pediatr..

[82] Li J., Lv L., Zhang J., Xu L., Zeng E., Zhang Z., Wang F., Tang X. (2019). A Combination of Peppermint Oil and Caraway Oil for the Treatment of Functional Dyspepsia: A Systematic Review and Meta-Analysis. Evid. Based Complement. Alternat. Med..

[83] Shavakhi A., Ardestani S.K., Taki M., Goli M., Keshteli A.H. (2012). Premedication with peppermint oil capsules in colonoscopy: A double blind placebo-controlled randomized trial study. Acta Gastroenterol. Belg..

[84] Khalaf M.H.G., Chowdhary S., Elmunzer B.J., Elias P.S., Castell D. (2019). Impact of Peppermint Therapy on Dysphagia and Non-cardiac Chest Pain: A Pilot Study. Dig. Dis. Sci..

[85] Akbari F., Rezaei M., Khatony A. (2019). Effect Of Peppermint Essence On The Pain And Anxiety Caused By Intravenous Catheterization In Cardiac Patients: A Randomized Controlled Trial. J. Pain Res..

[86] Borhani Haghighi A., Motazedian S., Rezaii R., Mohammadi F., Salarian L., Pourmokhtari M., Khodaei S., Vossoughi M., Miri R. (2010). Cutaneous application of menthol 10% solution as an abortive treatment of migraine without aura: A randomised, double-blind, placebo-controlled, crossed-over study. Int. J. Clin. Pract..

[87] Rafieian-Kopaei M., Hasanpour-Dehkordi A., Lorigooini Z., Deris F., Solati K., Mahdiyeh F. (2019). Comparing the Effect of Intranasal Lidocaine 4% with Peppermint Essential Oil Drop 1.5% on Migraine Attacks: A Double-Blind Clinical Trial. Int. J. Prev. Med..

[88] Göbel H., Heinze A., Heinze-Kuhn K., Göbel A., Göbel C. (2016). Peppermint oil in the acute treatment of tension-type headache. Schmerz.

[89] Davies S.J., Harding L.M., Baranowski A.P. (2002). A novel treatment of postherpetic neuralgia using peppermint oil. Clin. J. Pain.

[90] Akbari S.A., Alamolhoda S.H., Baghban A.A., Mirabi P. (2014). Effects of menthol essence and breast milk on the improvement of nipple fissures in breastfeeding women. J. Res. Med. Sci..

[91] Shanazi M., Farshbaf Khalili A., Kamalifard M., Asghari Jafarabadi M., Masoudin K., Esmaeli F. (2015). Comparison of the Effects of Lanolin, Peppermint, and Dexpanthenol Creams on Treatment of Traumatic Nipples in Breastfeeding Mothers. J. Caring Sci..

[92] Hoffman T. (2007). Ginger: An ancient remedy and modern miracle drug. Hawaii Med. J..

[93] Pagano E., Souto E.B., Durazzo A., Sharifi-Rad J., Lucarini M., Souto S.B., Salehi B., Zam W., Montanaro V., Lucariello G. (2020). Ginger (*Zingiber officinale* Roscoe) as a nutraceutical: Focus on the metabolic, analgesic, and antiinflammatory effects. Phytother. Res..

[94] Unuofin J.O., Masuku N.P., Paimo O.K., Lebelo S.L. (2021). Ginger from Farmyard to Town: Nutritional and Pharmacological Applications. Front. Pharmacol..

[95] Kiuchi F., Iwakami S., Shibuya M., Hanaoka F., Sankawa U. (1992). Inhibition of prostaglandin and leukotriene biosynthesis by gingerols and diarylheptanoids. Chem. Pharm. Bull..

[96] van Breemen R.B., Tao Y., Li W. (2011). Cyclooxygenase-2 inhibitors in ginger (*Zingiber officinale*). Fitoterapia.

[97] Gopalsamy B., Farouk A.A.O., Tengku Mohamad T.A.S., Sulaiman M.R., Perimal E.K. (2017). Antiallodynic and antihyperalgesic activities of zerumbone via the suppression of IL-1β, IL-6, and TNF-α in a mouse model of neuropathic pain. J. Pain Res..

[98] Hitomi S., Ono K., Terawaki K., Matsumoto C., Mizuno K., Yamaguchi K., Imai R., Omiya Y., Hattori T., Kase Y. (2017). [6]-gingerol and [6]-shogaol, active ingredients of the traditional Japanese medicine hangeshashinto, relief oral ulcerative mucositis-induced pain via action on Na(+) channels. Pharmacol. Res..

[99] Jeena K., Liju V.B., Kuttan R. (2013). Antioxidant, anti-inflammatory and antinociceptive activities of essential oil from ginger. Indian J. Physiol. Pharmacol..

[100] Mozafari S., Esmaeili S., Momenyan S., Zadeh Modarres S., Ozgoli G. (2021). Effect of *Zingiber officinale* Roscoe rhizome (ginger) capsule on postpartum pain: Double-blind randomized clinical trial. J. Res. Med. Sci..

[101] Ozgoli G., Goli M., Moattar F. (2009). Comparison of effects of ginger, mefenamic acid, and ibuprofen on pain in women with primary dysmenorrhea. J. Altern. Complement. Med..

[102] Daily J.W., Zhang X., Kim D.S., Park S. (2015). Efficacy of Ginger for Alleviating the Symptoms of Primary Dysmenorrhea: A Systematic Review and Meta-analysis of Randomized Clinical Trials. Pain Med..

[103] Cady R.K., Goldstein J., Nett R., Mitchell R., Beach M.E., Browning R. (2011). A double-blind placebo-controlled pilot study of sublingual feverfew and ginger (LipiGesic™ M) in the treatment of migraine. Headache.

[104] Therkleson T. (2014). Ginger Therapy for Osteoarthritis: A Typical Case. J. Holist Nurs..

[105] Al-Nahain A., Jahan R., Rahmatullah M. (2014). *Zingiber officinale*: A Potential Plant against Rheumatoid Arthritis. Arthritis.

[106] Di Cesare Mannelli L., Tenci B., Zanardelli M., Maidecchi A., Lugli A., Mattoli L., Ghelardini C. (2015). Widespread pain reliever profile of a flower extract of *Tanacetum parthenium*. Phytomedicine.

[107] Pareek A., Suthar M., Rathore G.S., Bansal V. (2011). Feverfew (*Tanacetum parthenium* L.): A systematic review. Pharmacogn Rev..

[108] Jain N.K., Kulkarni S.K. (1999). Antinociceptive and anti-inflammatory effects of *Tanacetum parthenium* L. extract in mice and rats. J. Ethnopharmacol..

[109] Galeotti N., Maidecchi A., Mattoli L., Burico M., Ghelardini C. (2014). St. John’s Wort seed and feverfew flower extracts relieve painful diabetic neuropathy in a rat model of diabetes. Fitoterapia.

[110] Johnson E.S., Kadam N.P., Hylands D.M., Hylands P.J. (1985). Efficacy of feverfew as prophylactic treatment of migraine. Br. Med. J..

[111] Murphy J.J., Heptinstall S., Mitchell J.R. (1988). Randomised double-blind placebo-controlled trial of feverfew in migraine prevention. Lancet.

[112] Saranitzky E., White C.M., Baker E.L., Baker W.L., Coleman C.I. (2009). Feverfew for migraine prophylaxis: A systematic review. J. Diet. Suppl..

[113] Urošević M., Nikolić L., Gajić I., Nikolić V., Dinić A., Miljković V. (2022). Curcumin: Biological Activities and Modern Pharmaceutical Forms. Antibiotics.

[114] Sun J., Chen F., Braun C., Zhou Y.Q., Rittner H., Tian Y.K., Cai X.Y., Ye D.W. (2018). Role of curcumin in the management of pathological pain. Phytomedicine.

[115] Razavi B.M., Ghasemzadeh Rahbardar M., Hosseinzadeh H. (2021). A review of therapeutic potentials of turmeric (*Curcuma longa*) and its active constituent, curcumin, on inflammatory disorders, pain, and their related patents. Phytother. Res..

[116] Lubbad A., Oriowo M.A., Khan I. (2009). Curcumin attenuates inflammation through inhibition of TLR-4 receptor in experimental colitis. Mol. Cell. Biochem..

[117] Srivastava S., Saksena A.K., Khattri S., Kumar S., Dagur R.S. (2016). *Curcuma longa* extract reduces inflammatory and oxidative stress biomarkers in osteoarthritis of knee: A four-month, double-blind, randomized, placebo-controlled trial. Inflammopharmacology.

[118] Shep D., Khanwelkar C., Gade P., Karad S. (2019). Safety and efficacy of curcumin versus diclofenac in knee osteoarthritis: A randomized open-label parallel-arm study. Trials.

[119] Jamali N., Adib-Hajbaghery M., Soleimani A. (2020). The effect of curcumin ointment on knee pain in older adults with osteoarthritis: A randomized placebo trial. BMC Complement. Med. Ther..

[120] Esmaeili F., Zahmatkeshan M., Yousefpoor Y., Alipanah H., Safari E., Osanloo M. (2022). Anti-inflammatory and anti-nociceptive effects of Cinnamon and Clove essential oils nanogels: An in vivo study. BMC Complement. Med. Ther..

[121] Haro-González J.N., Castillo-Herrera G.A., Martínez-Velázquez M., Espinosa-Andrews H. (2021). Clove Essential Oil (*Syzygium aromaticum* L. Myrtaceae): Extraction, Chemical Composition, Food Applications, and Essential Bioactivity for Human Health. Molecules.

[122] Booth N.L., Overk C.R., Yao P., Burdette J.E., Nikolic D., Chen S.N., Bolton J.L., van Breemen R.B., Pauli G.F., Farnsworth N.R. (2006). The chemical and biologic profile of a red clover (*Trifolium pratense* L.) phase II clinical extract. J. Altern. Complement. Med..

[123] Sabudak T., Guler N. (2009). *Trifolium* L.— review on its phytochemical and pharmacological profile. Phytother. Res..

[124] Booth N.L., Piersen C.E., Banuvar S., Geller S.E., Shulman L.P., Farnsworth N.R. (2006). Clinical studies of red clover (*Trifolium pratense*) dietary supplements in menopause: A literature review. Menopause.

[125] Ferraris C., Ballestra B., Listorti C., Cappelletti V., Reduzzi C., Scaperrotta G.P., Pulice I., Ferrari E.G.A., Folli S., Mariani L. (2020). Red clover and lifestyle changes to contrast menopausal symptoms in premenopausal patients with hormone-sensitive breast cancer receiving tamoxifen. Breast Cancer Res. Treat..

[126] Ghazanfarpour M., Sadeghi R., Roudsari R.L., Khorsand I., Khadivzadeh T., Muoio B. (2016). Red clover for treatment of hot flashes and menopausal symptoms: A systematic review.w and meta-analysis. J. Obstet. Gynaecol..

[127] Luís Â., Domingues F., Pereira L. (2018). Effects of red clover on perimenopausal and postmenopausal women’s blood lipid profile: A meta-analysis. Climacteric.

[128] Vishali N., Kamakshi K., Suresh S., Prakash S. (2011). Red clover *Trifolium pratense* (Linn.) isoflavones extract on the pain threshold of normal and ovariectomized rats—A long-term study. Phytother. Res..

[129] Wang X.S., Guan S.Y., Liu A., Yue J., Hu L.N., Zhang K., Yang L.K., Lu L., Tian Z., Zhao M.G. (2019). Anxiolytic effects of Formononetin in an inflammatory pain mouse model. Mol. Brain.

[130] Gartoulla P., Han M.M. (2014). Red clover extract for alleviating hot flushes in postmenopausal women: A meta-analysis. Maturitas.

[131] Berardino K., Carroll A.H., Popovsky D., Ricotti R., Civilette M.D., Sherman W.F., Kaye A.D. (2022). Opioid Use Consequences, Governmental Strategies, and Alternative Pain Control Techniques Following Total Hip Arthroplasties. Orthop Rev..

